# Benzenesulfonamide Analogs: Synthesis, Anti-GBM Activity and Pharmacoprofiling

**DOI:** 10.3390/ijms241512276

**Published:** 2023-07-31

**Authors:** Akshaya Murugesan, Saravanan Konda Mani, Ramesh Thiyagarajan, Suresh Palanivel, Atash V. Gurbanov, Fedor I. Zubkov, Meenakshisundaram Kandhavelu

**Affiliations:** 1 Department of Biotechnology, Lady Doak College, Madurai Kamaraj University, Thallakulam, Madurai 625002, India; akshaya.murugesan@tuni.fi; 2 Molecular Signaling Group, Faculty of Medicine and Health Technology, Tampere University and BioMediTech, P.O. Box 553, 33101 Tampere, Finland; sureshpalanivel21@gmail.com; 3 Department of Biotechnology, Bharath Institute of Higher Education & Research, Chennai 600073, India; saravananbioinform@gmail.com; 4 Department of Basic Medical Sciences, College of Medicine, Prince Sattam Bin Abdulaziz University, Al-Kharj 11942, Saudi Arabia; r.thiyagarjan@psau.edu.sa; 5 Centro de Química Estrutural, Institute of Molecular Sciences, Instituto Superior Técnico, Universidade de Lisboa, Av. Rovisco Pais, 1049-001 Lisboa, Portugal; organik10@hotmail.com; 6 Excellence Center, Baku State University, Z. Xalilov Str. 23, Az 1148 Baku, Azerbaijan; 7 Organic Chemistry Department, Faculty of Science, RUDN University, 6 Miklukho-Maklaya St., 117198 Moscow, Russia; fzubkov@sci.pfu.edu.ru

**Keywords:** benzenesulfonamide analogs, RTK inhibitor, glioblastoma, cell death, pharmacokinetics

## Abstract

The tropomyosin receptor kinase A (TrkA) family of receptor tyrosine kinases (RTKs) emerge as a potential target for glioblastoma (GBM) treatment. Benzenesulfonamide analogs were identified as kinase inhibitors possessing promising anticancer properties. In the present work, four known and two novel benzenesulfonamide derivatives were synthesized, and their inhibitory activities in TrkA overexpressing cells, U87 and MEF cells were investigated. The cytotoxic effect of benzenesulfonamide derivatives and cisplatin was determined using trypan blue exclusion assays. The mode of interaction of benzenesulfonamides with TrkA was predicted by docking and structural analysis. ADMET profiling was also performed for all compounds to calculate the drug likeness property. Appropriate QSAR models were developed for studying structure–activity relationships. Compound 4-[2-(4,4-dimethyl-2,6-dioxocyclohexylidene)hydrazinyl]-*N*-(5-methyl-1,3,4-thiadiazol-2-yl)benzenesulfon-amide (AL106) and 4-[2-(1,3-dioxo-1,3-dihydro-2*H*-inden-2-ylidene)hydrazinyl]-*N*-(5-methyl-1,3,4-thiadiazol-2-yl)benzenesulfonamide (AL107) showed acceptable binding energies with the active sites for human nerve growth factor receptor, TrkA. Here, AL106 was identified as a potential anti-GBM compound, with an IC_50_ value of 58.6 µM with a less toxic effect in non-cancerous cells than the known chemotherapeutic agent, cisplatin. In silico analysis indicated that AL106 formed prominent stabilizing hydrophobic interactions with Tyr359, Ser371, Ile374 and charged interactions with Gln369 of TrkA. Furthermore, in silico analysis of all benzenesulfonamide derivatives revealed that AL106 has good pharmacokinetics properties, drug likeness and toxicity profiles, suggesting the compound may be suitable for clinical trial. Thus, benzenesulfonamide analog, AL106 could potentially induce GBM cell death through its interaction with TrkA and might be an attractive strategy for developing a drug targeted therapy to treat glioblastoma.

## 1. Introduction

Glioblastoma multiforme (GBM), a grade 4 glial tumor is nourished by anomalous tumor blood supply vessels. Being infiltrative, GBM cells invade into nearby and opposite regions of the brain, but rarely spread outside of the brain. GBM acquires high intra tumoral heterogeneity due to the overexpression or mutations in MAPK signaling pathways including, receptor tyrosine kinases (RTKs) [1]. RTKs are involved in regulating cellular functions such as proliferation, differentiation, cell survival, metabolism, cell migration, and cell cycle control [2]. The most crucial RTKs include epidermal growth factor receptor (EGFR), insulin like growth factor receptor (IGFR), platelet-derived growth factor receptor (PDGFR), vascular endothelial growth factor (VEGF) and nerve growth factor receptor (NGFR), which are the current targets in developing cancer therapeutics particularly for GBM. In humans, the RTKs share an extracellular domain, trans-membrane helix and intracellular region [3].

Targeted molecular therapies have been the focus of therapies against GBM and other cancers where RTK targeted drugs possess a high capacity with reduced toxicity. Notably, tropomyosin receptor kinases (Trk receptors) are a family of membrane-bound tyrosine kinases that possess significant oncogenic roles. Out of three common types of Trk receptors, overexpression of TrkA encoded by NTRK1, promotes cell growth, migration and invasion in vitro. TrkA/B/C plays vital roles in pain response, movement, body temperature, memory and proprioception, and overexpression of Trk protein led to oncogenesis in various types of cancer [4]. TrkA serves as a high affinity receptor for the nerve growth factor (NGF), and therefore binding of a potential ligand results in downstream activation of Ras/MAP Kinase and PI3 Kinase pathways, which are closely associated with several cancers [5].

Novel Trk inhibitors, such as larotrectinib and entrectinib exhibit impressive clinical properties in cancer patients with Trk fusions. NTRK fusions were found to be the potential driver mutations in glioma. Considering the prognosis of GBM, an effective TrkA inhibitor is essential to regulate the downstream signaling pathway. Recently, TrkA targeting hydrazone derivatives were identified as biologically active drug molecules with pharmacological importance in treating GBM. The combinations of hydrazone and modified functional groups results in enhanced derivatives possessing various biological activities [6,7,8]. It was observed that benzimidazole derivatives exhibited many potential biological activities including anticancer, antidepressant, anti-inflammatory, antiplatelet, cardio protective etc. [9]. The addition of a heteroaryl and heteroacyl hydrazone moiety on the basic ring nucleus is potentially of interest, to obtain various biologically active compounds. We previously reported the effect of 23 novel arylhydrazones of active methylene derivatives against the growth of human brain astrocytoma cells and the results showed a better anti-proliferation effect at the micro molar level [10]. We have established compound R234 as a promising RTK inhibitor with anti-GBM potential [11]. We also noticed that the compounds with benzenesulfonate showed better interaction with the TrkA receptor with low anti-GBM potential [11] than the other hydrazone derivatives [12,13]. In the present work we have described the synthesis and structure–activity relationships of a series of benzenesulfonamide compounds with higher TrkA interaction potential.

## 2. Results 

### 2.1. Benzenesulfonamide Derivatives’ Interaction with Receptor Tyrosine Kinase, TrkA

**Figure 1:**
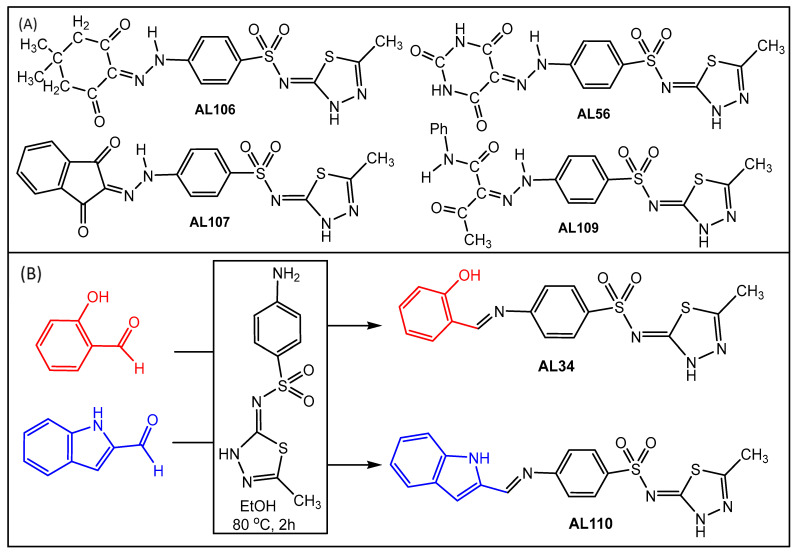
(**A**) Structures of benzenesulfonamide derivatives and (**B**) Synthesis scheme of Schiff bases.

Benzenesulfonamide derivatives AL56, AL106, AL107, AL109 (Figure 1A) AL34 and AL110 (Figure 1B) were synthesized as described in the Methods section. The purity of AL56, AL106, AL107, AL109 derivatives (>95%) were verified from the melting point, Nuclear Magnetic Resonance (NMR) spectrum, elemental analysis and mass spectrometry measurements as reported in [14,15]. The ^1^H-NMR spectra of AL34 and AL110 in DMSO-*d*_6_, and CH=N is observed at 10.25 and 9.93 ppm, respectively. In the IR spectra of AL34 and AL110, the stretching vibration of *ν*(C=N) was observed at 1627 cm^−1^ and 1630 cm^−1^, respectively. Elemental analysis and ESI-MSin methanol (peak at *m*/*z* 375.5 and 398.5) supported the proposed formulation of AL34 and AL110. Structures of all the derivatives are presented in Figure 1.

**Figure 2:**
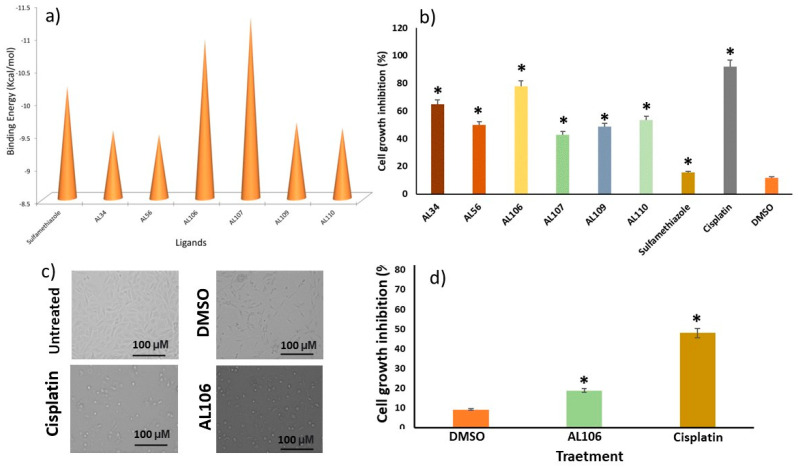
(**a**) Docking scores of a panel of novel benzenesulfonamide compounds with tyrosine kinase TrkA. (**b**) Cytotoxicity assay of benzenesulfonamide derivatives in U87 cell line. (**c**) Phase contrast image of U87 cells, untreated and DMSO, cisplatin AL106 treated. (**d**) Percentage of cell growth inhibition by AL106 and cisplatin in MEF cells. Cellular viability was measured by the trypan blue exclusion method. Datapoints and error bars represent mean ± S.E.M from *n* = 6 values per group and were analyzed by one-way ANOVA. * *p* < 0.05 denotes differences between DMSO versus treated conditions.

To obtain a deeper insight into the mode of action, the sulfonamide compound and its variants, along with newly developed benzenesulfonamide derivatives were docked with the human receptor tyrosine kinase, TrkA protein. The target protein 1HE7 was therefore co-crystallized with glycerol and the observed contacts in the crystal structure were used as a reference point. To choose the best-docked compounds, the Autodock Vina score value was used. Figure 2a shows the binding energy of the compounds with TrkA. The Autodock Vina scores of all the derivatives along with the TrkA are presented in Figure 2a. The binding energy of AL34, AL56, AL106, AL107, AL109, AL110 and sulfamethiazole were identified as −9.54, −9.48, −10.93, −11.26, −9.66, −9.58, and 10.21 Kcal/mol, respectively. These results (binding energy > −8.0 Kcal/mol) demonstrated that benzenesulfonamide derivatives have strong binding affinity with TrkA.

### 2.2. AL106 Effectively Reduces Cell Viability of GBM Cells

To validate the TrkA-induced cell death efficiency through the binding of benzenesulfonamide derivatives, the effect on cell proliferation and viability was assessed in GBM cells. The cell growth inhibition potential of all the compounds were determined using trypan blue exclusion method in U87 cells at 100 µM concentration. The compound AL106 was found to exhibit higher cytotoxic potential with 78% inhibition compared to the other derivatives (Figure 2b). The compounds AL34 and AL110 showed 64.7% and 53.3% inhibition, respectively, while other compounds showed less than 50% of cell growth inhibition. The control drug cisplatin exhibited 90% inhibition in U87 cells. Phase-contrast microscopy observations also validated the morphological changes in drug treated samples compared to the untreated cells. The microscopic representative images showed that compound AL106 and cisplatin treated cells had different morphologies with reduced size, while the DMSO and untreated cells appeared thin with flattened morphology and were well-attached in the growing environment (Figure 2c). Since our interest is to generalize the effect of synthesized benzenesulfonamide analogs on TrkA overexpressing non-cancerous cells, only the top compound, AL106 was selected for the cytotoxicity analysis. The cytotoxicity study in U87 and MEF cells revealed that AL106 induced significant cell death in GBM compared to non-tumorous cells. With a 10 μM treatment, ~40% growth inhibition was observed in U87 cells while <10% growth inhibition was observed in MEF (Figure 2d). These results suggest that the benzenesulfonamide derivative, AL106 induced a less cytotoxic effect in non-cancerous cells than the known chemotherapeutic agent, cisplatin. The data also suggest that AL106 can induce cell death through the receptor tyrosine kinase interaction in GBM cells. It is evident that AL106 possesses a higher anti-GBM effect and hence this compound was further tested for its cell growth inhibition in a dose-dependent manner. 

### 2.3. AL106 Effectively Reduces the GBM Proliferation

**Figure 3:**
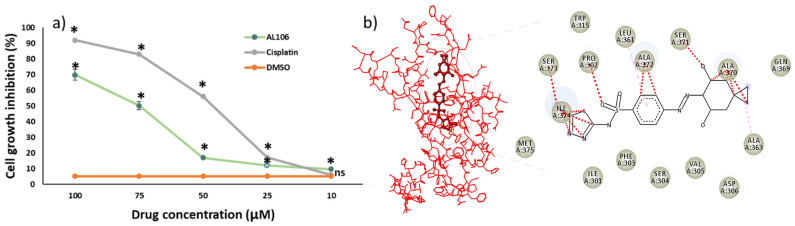
AL106–TrkA interaction inhibits GBM cell proliferation in a dose-dependent manner. (**a**) Percentage of GBM cell growth inhibition under DMSO, AL106 and cisplatin treated conditions for 24 h. (**b**) Structure–activity relationship of AL106 with TrkA receptor. Datapoints and error bars represent mean ± S.E.M (*n* = 6 independent values per group) using one-way ANOVA. * *p* < 0.05 indicates the statistical differences between DMSO and treated conditions. ns represents a non-statistically significant difference between DMSO and AL106 and cisplatin treated conditions.

U87 cells were treated with different concentrations of AL106, and cisplatin as described in the Methods section. The lead molecule AL106, potently reduced cell proliferation over the tested concentrations (Figure 3a). Among the different concentrations tested, 100 µM showed a higher cytotoxic effect. It was also noted that, as the concentration of the compound increased, the cell growth inhibition also increased, suggesting that AL106 induces cell death in a dose-dependent manner. The half maximal inhibitory concentration (IC_50_) was found to be 58.6 µM and 53 µM for AL106 and cisplatin, respectively. To explore the mode of interaction of AL106 with the TrkA target further, molecular docking was performed. AL106 formed stabilizing hydrophobic interactions with the amino acid residues Tyr_359_, Ser_371_, and Ile_374_. Charged interactions in the backbone atoms of Gln_369_ were observed to make close contacts with the ligand (Figure 3b). The residue Ala was identified as creating multiple interactions with some of the docked molecules. Small residues Ala_363_, Ala_364_, Ala_370_, Ala_372_, Gly_307_, Gly_368_, and Pro_302_ have also been found to form interactions with the target protein. Overall, these results indicated the importance of the charged amino acid residues and alanine residues at the interface that are required for binding to, and activating the receptor [16,17]. These results suggest that a stronger interaction between AL106 and TrkA might have induced cell death through the activation of the TrkA downstream signaling cascade in GBM cells. 

### 2.4. Benezesulfonamide Derived Compounds Exhibit Drug-like Properties 

Benezesulfonamide derived compounds, AL56, AL106, AL107, AL109, AL34, AL110 and sulfonamide were assessed for their drug-likeness. Various descriptors representing the drug-likeness of the compounds are tabulated in Table 1**.** Based on the analysis, it was found that the topological polar surface area (TPSA) was higher for AL56 with 208.23 and lower for sulfamethiazole with 134.59. TPSA is an efficient method to identify the mechanism of action and the nature of molecular interactions between drug and the target protein. Likewise, HBD/HBA which is an important descriptor for drug–target binding, was found to be higher for AL56, with a value of 8, compared to the other compounds. Additionally, human intestinal absorption (HIA), an important factor in pharmacokinetics, was found to be comparatively higher for sulfamethiazole than the other compounds studied. AL110 was found to be an inhibitor of Pgp substrate, CYP1A2, CYP2C19, CYP2C9 and CYP3A4, which are important features to take into consideration for drug interactions. All the selected compounds have a bioavailability score of 0.55 and lipophilicity (xlogP3) in the recommended range between 0 to 6. All the compounds have ‘0’ violation of Lipinski’s rule, except AL56 which has the least accepted violation of Lipinski’s rule, and log *p* value < 5, thus displaying a drug likeness for compounds for oral administration. However, further study is still warranted at the clinical level.

### 2.5. Quantitative Structure–Activity Relationship (QSAR) Analysis of Synthesized Derivatives and Their Biological Activity 

**Figure 4:**
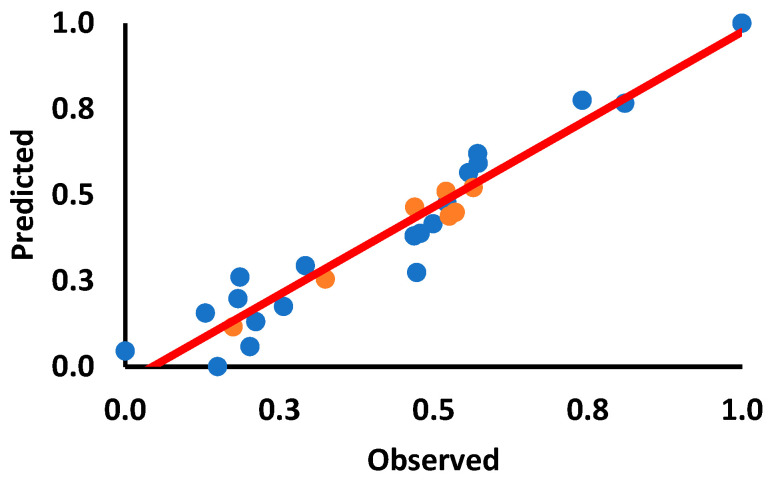
Graphical plot between observed and predicted activity values of the training (blue dots) and test sets of benzenesulfonamide derivatives (orange dots).

We subsequently determined the biological potential of the benzenesulfonamide derivatives, AL56, AL106, AL107, AL109, AL34, AL110 and sulfonamide using the AutoQSAR model. It was observed that the compounds showed a strong correlation for the training and test set (Table 2) and were proven to have good biological activity. The kernel partial least squares regression (KPLS) model was used to generate the QSAR model, in which the compounds were considered outliers with a significant deviation from the regression line. The correlation coefficient value (r) was used to cross-validate the QSAR model’s validity and predictability for the anticancer activity. The correlation coefficient values (r) are 0.8385 for the training set and 0.8282 for the test set (Figure 4). These data show the effectiveness of the model developed, and the observations made it very evident that the compound predicted was a potent anticancer agent. The structure–activity relationship of this series of benzenesulfonamides, including the sulphonamides exhibited better cytotoxic effect than the structurally similar compounds, TrkA inhibitors. Specifically, AL106 showed strong correlation with the model, supporting its toxic effect on cancer cells, suggesting that AL106 has the potential to be developed as a drug candidate for treating GBM.

## 3. Discussion

Glioblastoma drug discovery involves complex processes including target identification, optimization, pre-clinical and clinical trials [18,19,20,21,22]. Although several drugs have been developed for treating GBM, many of them failed at the clinical phase trial [23]. In recent decades, many synthetic compounds are being developed to target TrkA receptors for treating GBM. The TrkA pathway is one of the most important signaling pathways that involves tumor growth and differentiation. Notably, Entrectinib, a TrkA inhibitor has been used for the treatment of GBM and brain metastases and it possesses blood–brain barrier crossing potential. However, Entrectinib induced many side effects including anemia, breathing difficulties, constipation, dizziness, diarrhea, edema, fatigue, taste disorders, nausea, paresthesia, and weight gain. The recently developed TrkA pathway targeting drug Tandutinib, has also been discontinued in Phase 2 for treating GBM, thus demand for developing new Trk targeted compound is high.

The present investigation revealed that the newly developed benzene derivative AL106, targeting TrkA exhibited a higher cytotoxic effect in the U87, GBM cell line than the other synthesized compounds. Our previous studies also revealed the structurally similar hydrazone derivative, R234 to be a TrkA inhibitor by downregulating cyclins, cyclin-dependent kinases and other key molecules involved in the cell cycle. Meanwhile, at a concentration of 100 μM, the cytotoxic effect of R234 was found to be around 45%, which is less than AL106, which exhibits 80%. Additionally, the GLIDE score for R234 was found to be −4.12 whereas AL106 had −11, thus indicating the strong binding of AL106 with the TrkA receptor compared to the known TrkA inhibitor R234. The IC_50_ value obtained from the dose–response curve revealed a similar trend for both AL106 and cisplatin. Our results are in agreement with previous studies [24,25,26] where the benzene sulphonamide derived compounds exhibited potent antitumor activity and it could be inferred that AL106 may have interacted with TrkA, potentiality targeting the EGFR signaling pathway. This observation is also supported by several previous studies on similar derivatives as potent anticancer agents [11]. 

The majority of drug failures are due to inadequate information for the in vitro ADMET properties [27]. The present study involved profiling the in silico ADMET properties for all chemically synthesized sulfamethizole derivatives where the drug absorption rate of HIA, CaCO_2_ permeability, CNS and BBB permeability was measured. Highly polar compounds do not cross the blood–brain barrier, yet these chemically synthesized compounds with high hydrophilicity can be modified to become lipophilic to enhance the BBB permeability. Our synthesized compounds showed lower HIA absorption rates, while sulfamethizole possesses a high HIA absorption rate suggesting sulfamethizole is a better bioactive compound for oral administration. Thus, the newly synthesized compound AL106, could be considered as a potential RTK inhibitor for the development of an anti-GBM drug. Many early-stage therapies fail due to the difficulty in predicting ADMET properties for a number of reasons. The quantity and chemical diversity of experimental data are often constrained, and the accuracy of predictions vary greatly. Furthermore, ADMET properties, including oral bioavailability, HIA, and metabolic stability, result from several physiological pathways, making it challenging to characterize them using conventional prediction models. It is imperative to acknowledge the limitations of ADMET prediction since the study does not include experimental confirmation. 

## 4. Materials and Methodology

### 4.1. Chemistry

A series of benzenesulfonamide derivatives, *N*-(5-methyl-1,3,4-thiadiazol-2-yl)-4-[2-(2,4,6-trioxotetrahydro-5(2*H*)-pyrimidinylidene)hydrazinyl]benzenesulfonamide (**AL56**), 4-[2-(4,4-dimethyl-2,6-dioxocyclohexylidene)hydrazinyl]-*N*-(5-methyl-1,3,4-thiadiazol-2-yl)benzenesulfon-amide (**AL106**), 4-[2-(1,3-dioxo-1,3-dihydro-2*H*-inden-2-ylidene)hydrazinyl]-*N*-(5-methyl-1,3,4-thiadiazol-2-yl)benzenesulfonamide (**AL107**) and 2-(2-{4-[(5-methyl-1,3,4-thiadiazol-2-yl)sulfamoyl]phenyl}hydrazinylidene)-3-oxo-*N*-phenylbutanamide (**AL109**) (Figure 1A) were reported earlier [1,2], and hence will not be discussed herein. The 4-[(2-hydroxybenzylidene)amino]-*N*-(5-methyl-1,3,4-thiadiazol-2-yl)benzenesulfonamide (**AL34**) and 4-[2-(2,3-dihydro-1*H*-indol-3-ylmethylidene)hydrazinyl]-*N*-(5-methyl-1,3,4-thiadiazol-2-yl)benzenesulfonamide (**AL110**) were synthesized by the Schiff base condensation of (*Z*)-4-amino-*N*-(5-methyl-1,3,4-thiadiazol-2(3*H*)-ylidene)benzenesulfonamide with 2-hydroxybenzaldehyde or 1*H*-indole-2-carbaldehyde, respectively, at 80 °C in ethanol (Figure 1).

### 4.2. Synthesis of Novel Benzenesulfonamide Derivatives

The derivatives, AL56, AL106, AL107, AL109 were synthesized and reported previously [14,15]. ^1^H/^13^C NMR spectra of four known, AL56, AL106, AL107, AL109 and two new compounds, AL34 and AL110, were reported in the Supplementary File. The synthesis of AL34 and AL110 is described below.

A 1:1 equimolar methanolic solution of (*Z*)-4-amino-*N*-(5-methyl-1,3,4-thiadiazol-2(3*H*)-ylidene)benzenesulfonamide (0.540 g, 2 mmol) and 2-hydroxybenzaldehyde (0.244 mL, 2 mmol) (or 1H-indole-2-carbaldehyde (0.290 g, 2 mmol) for AL110) was slightly heated (80 °C) for 2 h with stirring. The characteristic yellow precipitate obtained by the Schiff condensation was filtered off and recrystallized from methanol.

**AL34**: Yield, 82%, soluble in methanol, ethanol, acetone and DMF. Anal. Calcd for C_16_H_14_N_4_O_3_S_2_ (*M* = 374.43): C, 51.32; H, 3.77; N, 14.96. Found: C, 51.30; H, 3.75; N, 14.75%. IR, cm^−1^: 3356 (s br.) *ν*(OH), 3052 (s br.) *ν*(NH), 1601 and 1627 *ν*(C=N). ESI-MS: *m*/*z*: 375.5 [M + H]^+^. ^1^H-NMRin DMSO-*d*_6_, *δ* (ppm): 1.03–1.07 (3H, CH_3_) 6.57–8.95 (8H, Ar–H), 10.25 (s, 1H, CH=N), 10.73 (s, 1H, O–H), 12.57 (s, 1H, N–H). ^13^C-{^1^H} NMRin DMSO-*d*_6_, *δ* (ppm): 16.1 (CH_3_), 112.6, 116.8, 117.3, 119.6, 122.3 and 127.8 (Ar–H), 129.3 (C_Ar_–CH=N), 132.6 (C–S), 136.5 (CH=N–C_Ar_), 139.7 (C_Ar_–OH), 151.9 and 154.7 (C=N).

**AL110**: Yield, 73%, soluble in methanol, ethanol, acetone and DMF. Anal. Calcd for C_18_H_15_N_5_O_2_S_2_ (*M* = 397.47): C, 54.39; H, 3.80; N, 17.62. Found: C, 54.31; H, 3.77; N, 17.56%. IR, cm^−1^: 3120 and 3041 (s br.) *ν*(NH), 1611 and 1630 *ν*(C=N). ESI-MS: *m*/*z*: 398.5 [M + H]^+^. ^1^H-NMRin DMSO-*d*_6_, *δ* (ppm): 1.62 (3H, CH_3_) 6.92–8.42 (8H, Ar–H), 9.56 (C–H of 1*H*-indole) 9.93 (s, 1H, CH=N), 12.27 (s, 1H, N–H) and 13.81 (s, 1H, N–H). ^13^C-{^1^H} NMRin DMSO-*d*_6_, *δ* (ppm): 16.2 (CH_3_), 112.5, 114.1, 118.2, 120.9, 122.2, 123.5 and 124.2 (C–H), 127.4 (C–CH=N), 127.7 (C–NH), 137.3 (C–C_indole_), 138.6 (C–S), 140.5 (CH=N–C_Ar_), 154.2 and 154.9 (C=N). 

### 4.3. Characterization of Synthesized Benzenesulfonamide Derivatives

The ^1^H and ^13^C NMR spectra were recorded at room temperature on a Bruker Avance II + 300 (UltraShield^™^ Magnet) spectrometer operating at 300.130 and 75.468 MHz for proton and carbon-13, respectively. The chemical shifts are reported in ppm using tetramethylsilane as the internal reference. The infrared spectra (4000–400 cm^−1^) were recorded on a BIO-RAD FTS 3000MX instrument in KBr pellets. Carbon, hydrogen, and nitrogen elemental analysis was carried out in a PerkinElmer 2400 analyzer. Electrospray mass spectra (ESI-MS) was performed with an ion-trap instrument (Varian 500-MS LC Ion Trap Mass Spectrometer) equipped with an electrospray ion source. For electrospray ionization, the drying gas and flow rate were optimized according to the particular sample with 35 p.s.i. nebulizer pressure. Scanning was performed from *m*/*z* 50 to 1000 in a methanol solution. The compounds were observed in the negative or positive mode (capillary voltage = 80–105 V). 

### 4.4. Computational Assessment of Benzenesulfonamide Derivatives–TrkA Interaction

The atomic coordinates of human nerve growth factor receptor TrkA were retrieved from the protein databank (PDB ID: 1HE7) with an atomic resolution of 2.00 Å [25]. The Autodock Tools were used to prepare the crystal structure for molecular docking [25]. The protein molecule was loaded into the workspace, and the missing side chains and loops were constructed, and the protein structure’s hydrogen bonds were optimized. The water molecules below 3 Å were removed. The energy of the structure was confirmed by subjecting the optimized crystal structure to minimization. The OPLS 2005 force field was used in all the procedures. Grid generation was accomplished by the usage of ligand binding residues in the crystal structure. The Autodock tool was used in the ligand preparation procedure of these derivatives. The hydrogen bonds were inserted, and the bond length was calculated using the OPLS 2005 program [26]. Autodock Vina was used to perform molecular docking where ten poses for each ligand were obtained and stored in an acceptable format for subsequent analysis. The protein–ligand interactions were plotted using the Discovery Studio visualizer [28].

### 4.5. Chemicals and Drug Preparation

Dulbecco’s Modified Eagle Medium-high glucose (DMEM) (Biowest, #L0102-500), Fetal Bovine Serum (FBS) (#S181H-500, Biowest, Nuaille, France), penicillin, streptomycin (Sigma-Aldrich, #P4333, St. Louis, MO, USA), ampicillin B (Sigma-Aldrich, #A9528m), dimethyl sulfoxide (DMSO, Sigma-Aldrich). The chemically synthesized benzenesulfonamide derivatives and known chemotherapeutic agent, cisplatin were dissolved in DMSO to obtain a stock solution of 100 mM. Intermediate dilutions were prepared using a stock solution for further experimental validation. 

### 4.6. Cell Culture

The human glioblastoma cell line, U87 (kind gift from Dr. Kirsi Granberg, Faculty of Medicine and Health Technology, Tampere, Finland) and the non-cancer cell, mouse embryonic fibroblast cell line (MEF, gifted by Prof. Pasi Kallio, Faculty of Medicine and Health Technology, Tampere, Finland) were selected for this study as they overexpress TrkA receptor protein [29,30]. These cells were grown in DMEM supplemented with 10% FBS and were used to investigate the anticancer effect of benzenesulfonamide derivatives. The medium was supplemented with 2mM sodium pyruvate, 1% penicillin–streptomycin and 0.025 mg/mL amphotericin B. The cells were grown to 70–80% confluency at 37 °C with 5% CO_2_ in a humidified incubator. 

### 4.7. Cytotoxic Effect of Benzenesulfonamide Derivatives

The cytotoxic effects of benzenesulfonamide derivatives were determined in GBM and MEF cells. The initial density of 1 × 10^5^ cells/well were seeded in a six-well plate and treated with 100 µM concentration of the derivatives or cisplatin, and incubated for 48 h. Cisplatin and DMSO (0.1%) were used, respectively, as the positive control (PC) and negative control. Treated cells were trypsinized and harvested by centrifugation at 153× *g* for 10 min. The collected cells were stained with trypan blue dye. The cells were loaded on a Countess II FL hemocytometer (Thermo Fisher Scientific, Carlsbad, CA, USA) where live and dead cell populations were quantified to study the effect of the drug treatment. Biological repeats were used to obtain reproducible data and the percentage of cell growth inhibition was calculated using the following equation.

(1) $$\mathrm{Cell}\;\mathrm{growth}\;\mathrm{inhibition}\left(\%\right)=\frac{Mean\;No.\;ofuntreated\;cells\;\left(DMSOcontrol\right)-Mean\;No.\;of\;treated\;cells}{Mean\;No.\;of\;untreated\;cells\;\left(DMSO\;control\right)}\times 100$$

### 4.8. Dose-Dependent Cell Viability Assay

The cytotoxicity assay was performed to determine the cell growth inhibition of the compounds on GBM cells. The cells were seeded at a density of 1 × 10^5^ cells/well in six-well plates and incubated for 24 h. The cells were then treated with 10 µM, 25 µM, 50 µM, 75 µM, and 100µM concentrations of top benzenesulfonamide derivatives and cisplatin. The DMSO (0.1%) and cisplatin served as negative and positive drug controls, respectively. Treated cells were harvested by centrifugation at 153× *g* for 10 min. The live and dead cells were stained using trypan blue and were counted using a Countess II FL hemocytometer. The percentage of cell growth inhibition was calculated using Equation (1). The half-maximal inhibitory concentrations (IC_50_) of these compounds and cisplatin were calculated using the dose–response curve.

### 4.9. ADMET and Drug-likeness Prediction

The absorption, distribution, metabolism, excretion, toxicity and drug-likeness properties were analyzed for the synthesized benzenesulfonamide derivatives. All the compounds used in this study were converted into canonical smiles and assessed for ADMET prediction using SwissADME (http://www.swissadme.ch/; accessed on 14 April 2023) and pkCSM (https://biosig.lab.uq.edu.au/pkcsm/prediction; accessed on 14 April 2023) online software tools. We analyzed physiochemical descriptors like water solubility, topological polar surface area, H-bond acceptors, H-bond donors, water solubility, and ADME parameters, pharmacokinetic properties, druglike nature [31] and medicinal chemistry friendliness of the drug. Other parameters like percentage of human intestinal absorption (HIA), CaCO_2_ permeability, P-glycoprotein substrate/inhibitor, BBB permeability, CYP substrate and inhibitor, AMES toxicity, renal OCT2 substrate, maximum tolerated dose in human (log mg/kg/day), oral rat acute toxicity (LD_50_) (mol/kg) and hepatotoxicity were predicted for benzenesulfonamide derivatives using Lipinski’s rule of five, as significant in determining the bioavailability of the molecules.

### 4.10. Quantitative Structure–Activity Relationship Models (QSAR) for AL106

QSAR analysis was performed with the all seven synthesized compounds and in addition another 20 compounds were selected that showed structural similarities [11] with AL106. In detail, Kingdraw was used to draw the structures of the synthesized ligands and generate the respective SMILES formula’s for the compounds. The Ligprep tool in Maestro was used to convert the three-dimensional structures and optimize them. For each ligand, Ligprep is used to create tautomers, optimize, and neutralize the charge. The default settings were all retained, and pH 7.0 was chosen. Utilizing the OPLS 2005 force field, partial atomic charges were calculated and the energy of the ligands was minimized. AutoQSAR was used to predict the relationship between biological activity and molecular descriptors (Maestro version 13.2.128, Schrodinger, LLC, New York, NY, USA). The molecular descriptors were automatically calculated in AutoQSAR and KPLS models were built. AutoQSAR was used to eliminate variables. The F-test (F), the square of cross regression (q2), the square of coefficient regression (R2), and standard deviation were used to evaluate the correlation’s quality (Std), where “n” stands for the number of compounds, while “r” stands for the correlation coefficient.

### 4.11. Data and Statistical Analysis

The statistical analysis was performed only for studies where each group size was *n* = 6 independent values. The data were represented as mean ± standard error. One-way analysis of variance using Dunnett’s multiple comparison test was performed to assess the statistical significance between the groups where, *p* < 0.05 was considered statistically significant.

## Figures and Tables

**Table 1:** Predicted ADMET characteristics of benzenesulfonamide analogs.

ADMET	Descriptors	AL34	AL56	AL106	AL107	AL109	AL110	Sulfamethiazole
Physicochemical Properties	H-bond acceptors	6	8	7	7	7	5	4
	H-bond donors	2	4	2	2	3	3	2
	TPSA	141.16	208.23	167.1	167.1	179.13	144.99	134.59
Lipophilicity	iLOGP	1.78	−0.26	1.54	1.65	1.6	1.54	1.5
	XLOGP3	2.17	0.44	1.76	2.32	3.42	2.97	0.54
	WLOGP	3.99	−0.28	3.07	2.02	3.15	3.38	2.13
	MLOGP	1.04	−1.01	0.33	0.22	0.5	1.55	−0.05
	Silicos-IT Log P	3.02	0.01	2.72	3.14	2.07	2.48	0.92
	Consensus Log P	2.4	−0.22	1.88	1.87	2.15	2.38	1.01
Water Solubility	ESOL Log S	−3.7	−2.63	−3.52	−4.13	−4.65	−4.33	−2.14
	ESOL Class	Soluble	Soluble	Soluble	Moderately soluble	Moderately soluble	Moderately soluble	Soluble
	Ali Log S	−4.77	−4.38	−4.89	−5.47	−6.86	−5.68	−2.94
	Ali Class	Moderately soluble	Moderately soluble	Moderately soluble	Moderately soluble	Poorly soluble	Moderately soluble	Soluble
	Silicos-IT LogSw	−5.78	−4.68	−6	−7.38	−6.67	−6.75	−3.44
	Silicos-IT class	Moderately soluble	Moderately soluble	Moderately soluble	Poorly soluble	Poorly soluble	Poorly soluble	Soluble
Pharmacokinetics	GI absorption	Low	Low	Low	Low	Low	Low	High
	BBB permeant	No	No	No	No	No	No	No
	Pgp substrate	No	No	No	No	No	Yes	No
	CYP1A2 inhibitor	No	No	No	No	No	Yes	No
	CYP2C19 inhibitor	No	No	No	Yes	No	Yes	No
	CYP2C9 inhibitor	Yes	No	No	No	Yes	Yes	No
	CYP2D6 inhibitor	No	No	No	No	No	No	No
	CYP3A4 inhibitor	No	No	No	Yes	Yes	Yes	No
	log Kp (cm/s)	−7.04	−8.48	−7.62	−7.26	−6.67	−6.72	−7.57
Druglikeness	Lipinski violations	0	1	0	0	0	0	0
	Bioavailability Score	0.55	0.55	0.55	0.55	0.55	0.55	0.55

**Table 2:** List of training set and test set compounds of benzenesulfonamide derivatives used in the QSAR model. The scaled observed and predicted values of the kernel partial least squares regression (KPLS) model in AUOQSAR model against the TrkA are also shown.

Training Set				
Sl. No	Compound	Chemical Name	Observed	Predicted
1	AL56	(*Z*)-*N*-(5-methyl-1,3,4-thiadiazol-2(3*H*)-ylidene)-4-(2-(2,4,6-trioxotetrahydropyrimidin-5(2*H*)-ylidene)hydrazineyl)benzenesulfonamide	0.2920	0.2939
2	AL107	(*Z*)-4-(2-(1,3-dioxo-1,3-dihydro-2*H*-inden-2-ylidene)hydrazineyl)-*N*-(5-methyl-1,3,4-thiadiazol-2(3*H*)-ylidene)benzenesulfonamide	0.2569	0.1753
3	AL109	(*Z*)-2-(2-(4-(*N*-((*Z*)-5-methyl-1,3,4-thiadiazol-2(3*H*)-ylidene)sulfamoyl)phenyl)hydrazineylidene)-3-oxo-*N*-phenylbutanamide	1.0000	1.0000
4	AL110	4-(((*E*)-indolin-2-ylmethylene)amino)-*N*-((*Z*)-5-methyl-1,3,4-thiadiazol-2(3*H*)-ylidene)benzenesulfonamide	0.5220	0.4761
5	R8	3-(2-(4-bromophenyl)hydrazineylidene) pentane-2,4-dione	0.4786	0.3883
6	R9	3-(2- (4-bromophenyl)hydrazineylidene) pentane-2,4-dione	0.4683	0.3806
7	R10	(2-(2-(2,4- dioxopentan-3-ylidene)hydrazineyl) phenyl)arsonic acid	0.8102	0.7668
8	R31	5-chloro-3-(2-(1-ethoxy-1,3-dioxobutan-2-ylidene)hydrazineyl)- 2-hydroxybenzenesulfonic acid	0.7412	0.7758
9	R40	ethyl 2-(2-(4-chlorophenyl)hydrazineylidene)-3- oxobutanoate	0.2023	0.0586
10	R46	5-(2-(1,3-dioxo-1-phenylbutan-2- ylidene)hydrazineyl)-4-hydroxybenzene-1,3-disulfonic acid	0.5571	0.5651
11	R156	4-(2-(2,4,6-trioxotetrahydropyrimidin- 5(2*H*)-ylidene)hydrazineyl)benzoic acid	0.5725	0.5923
12	R221	*N*,*N*-dimethyl-2-(2-(4-nitrophenyl)hydrazineylidene)-3- oxobutanamide	0.2119	0.1312
13	R235	3-(2-(2-nitrophenyl) hydrazineylidene)pentane-2,4-dione	0.1831	0.1977
14	R243	ethyl 2-(2-(4-cyanophenyl) hydrazineylidene)-3-oxobutanoate	0.0000	0.0456
15	R236	2-(2-(2,4-dioxopentan-3- ylidene)hydrazineyl) benzoic acid	0.1862	0.2609
16	R237	2-(2-(2,4-dioxopentan-3-ylidene)hydrazineyl) benzenesulfonic acid	0.1497	0.0000
17	R241	4-(2-(2,4-dioxopentan-3-ylidene)hydrazineyl) benzonitrile	0.1301	0.1559
18	R244	2-(2-(4,4-dimethyl-2,6-dioxocyclohexylidene) hydrazineyl) benzoic acid	0.4727	0.2739
19	R246	sodium 2-(2- (1,3-dioxo-1,3-diphenylpropan-2-ylidene)hydrazineyl)benzenesulfonate	0.5716	0.6203
20	R283	5-(2-(2,4- dioxopentan-3-ylidene)hydrazineyl)isophthalic acid	0.4994	0.4164
Test Set				
Sl. No	Compound	Chemical Name		
1	AL34	4-(((*E*)-2-hydroxybenzylidene)amino)-*N*-((*Z*)-5-methyl-1,3,4-thiadiazol-2(3*H*)-ylidene)benzenesulfonamide	0.5644	0.5212
2	AL106	(*Z*)-4-(2-(4,4-dimethyl-2,6-dioxocyclohexylidene)hydrazineyl)-*N*-(5-methyl-1,3,4-thiadiazol-2(3*H*)-ylidene)benzenesulfonamide	0.1750	0.1162
3	R2	3-(2-(2,4-dioxopentan-3-ylidene)hydrazineyl)-2- hydroxy-5-nitrobenzenesulfonic acid	0.4695	0.4641
4	R212	3-(2-(1-(dimethylamino)-1,3-dioxobutan-2- ylidene)hydrazineyl)-2-hydroxy-5-nitrobenzenesulfonic acid	0.3247	0.2548
5	R234	2-(2-(2,4-dioxopentan-3-ylidene) hydrazineyl)benzonitrile	0.5204	0.5103
6	R313	4-(2-(1-cyano-2-methoxy-2- oxoethylidene) hydrazineyl)benzoic acid	0.5352	0.4491
7	Sul	4-amino-*N*-(5-methyl-1,3,4-thiadiazol-2-yl)-benzenesulfonamide	0.5257	0.4384

## Data Availability

The data are available from the corresponding authors upon request.

## Supplementary Materials

The supporting information can be downloaded at: https://www.mdpi.com/article/10.3390/ijms241512276/s1.
