# Supplementary material for: Benzenesulfonamide Analogs: Synthesis, Anti-GBM Activity and Pharmacoprofiling
Source: Int J Mol Sci. 2023 Jul 31;24(15):12276. doi: 10.3390/ijms241512276 (PMC10418358; doi:10.3390/ijms241512276)
Supplement: Supplementary file 1 [file ijms-24-12276-s001.zip › ijms-2393453-supplementary.pdf]

Electronic Supporting Information

Benzenesulfonamide anologs: Synthesis, anti-GBM activity and pharmacoprofiling

Akshaya Murugesan[1,2], Saravanan Konda Mani[3], Thiyagarajan Ramesh[4], Suresh Planivel[2], Atash V. Gurbanov,[5,6] Fedor I. Zubkov[7] , and Meenakshisundaram Kandhavelu[2,*]

[1] Department of Biotechnology, Lady Doak College, Madurai Kamaraj University, Thallakulam, Madurai – 625002, India.
[2] Molecular Signaling Group, Faculty of Medicine and Health Technology, Tampere University and BioMediTech, P.O. Box 553, 33101 Tampere, Finland.
[3] Department of Biotechnology, Bharath Institute of Higher Education & Research, Chennai - 600 073, Tamilnadu, India.
[4] Department of Basic Medical Sciences, College of Medicine, Prince Sattam Bin Abdulaziz University, Al-Kharj, 11942, Kingdom of Saudi Arabia.
[5] Centro de Química Estrutural, Institute of Molecular Sciences, Instituto Superior Técnico, Universidade de Lisboa, Av. Rovisco Pais, 1049-001 Lisboa, Portugal
[6] Excellence Center, Baku State University, Z. Xalilov Str. 23, Az 1148 Baku, Azerbaijan
[7] Organic Chemistry Department, Faculty of Science, RUDN University, 6 Miklukho-Maklaya St, Moscow 117198, RussianFederation
* Correspondence: author: meenakshisundaram.kandhavelu@tuni.fi

1. Synthesis of arylhydrazones

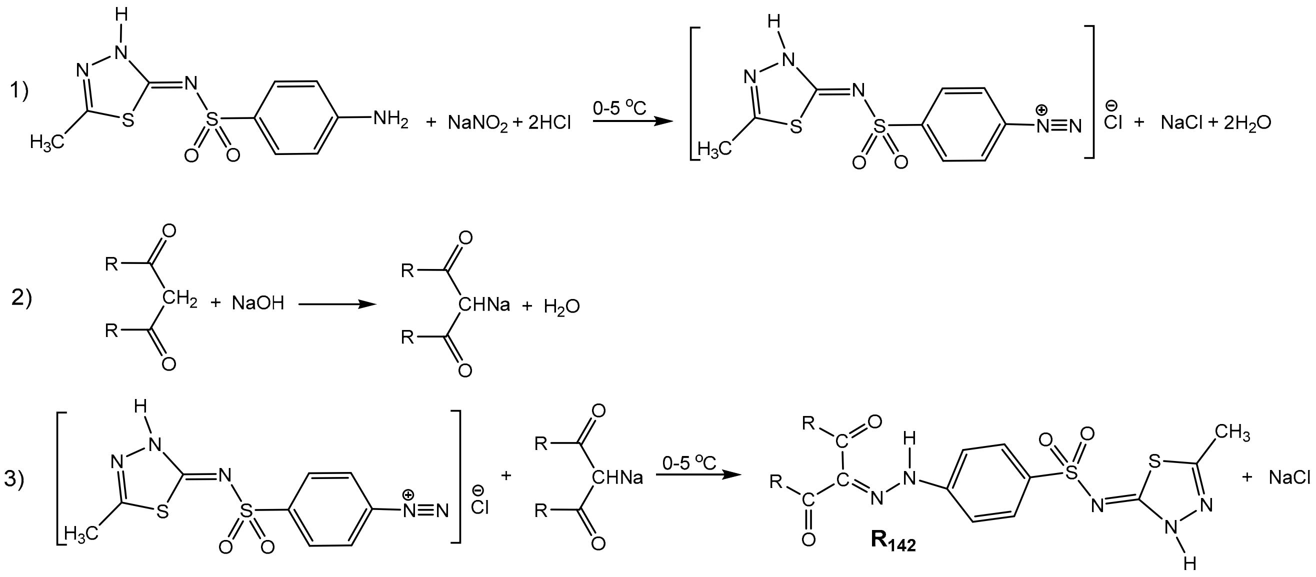

Scheme

2. $^{1}H/^{13}C$ NMR spectra of arylhydrazones

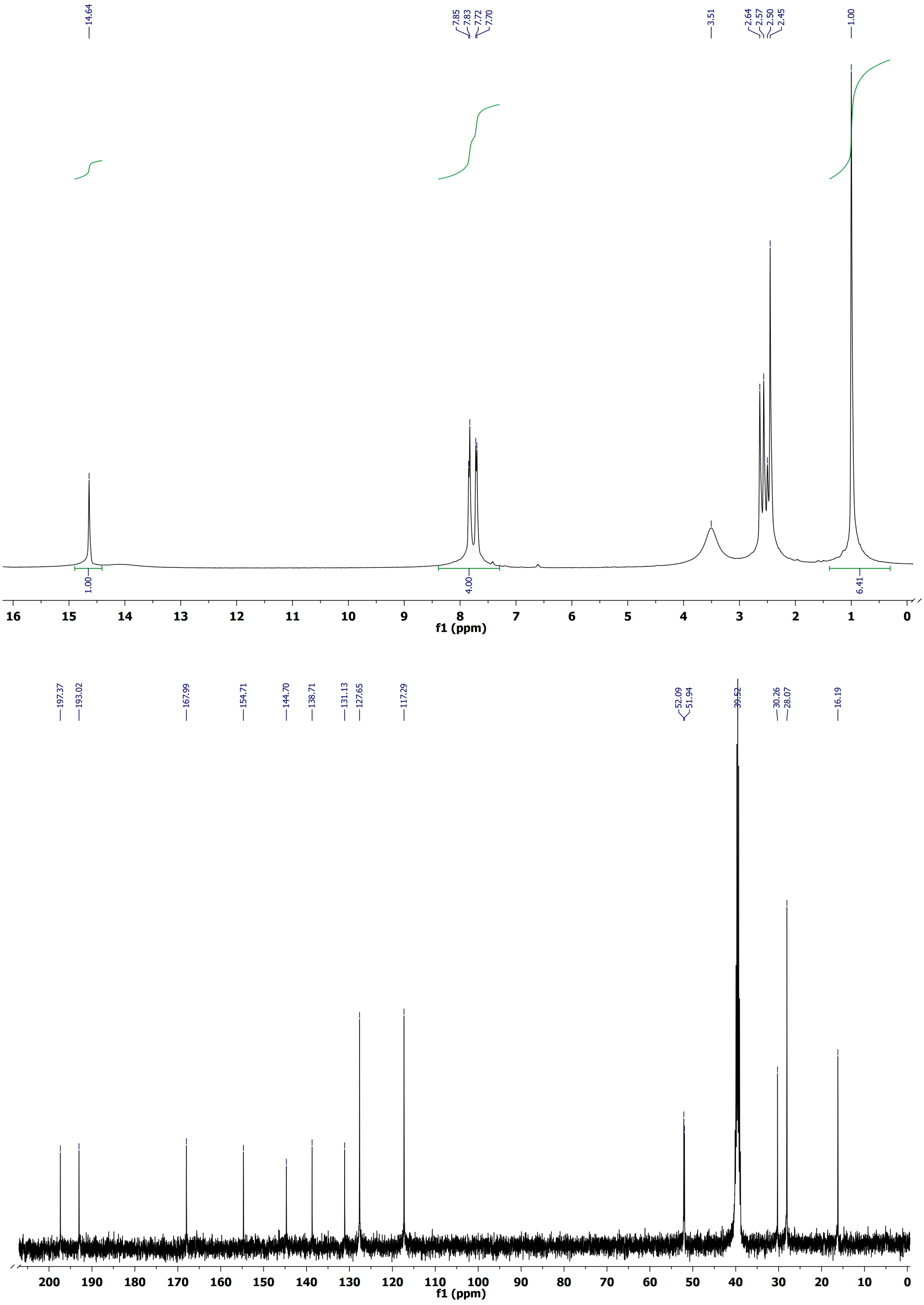

Figure S1. $^{1}H/^{13}C$ NMR spectra of **AL106.**

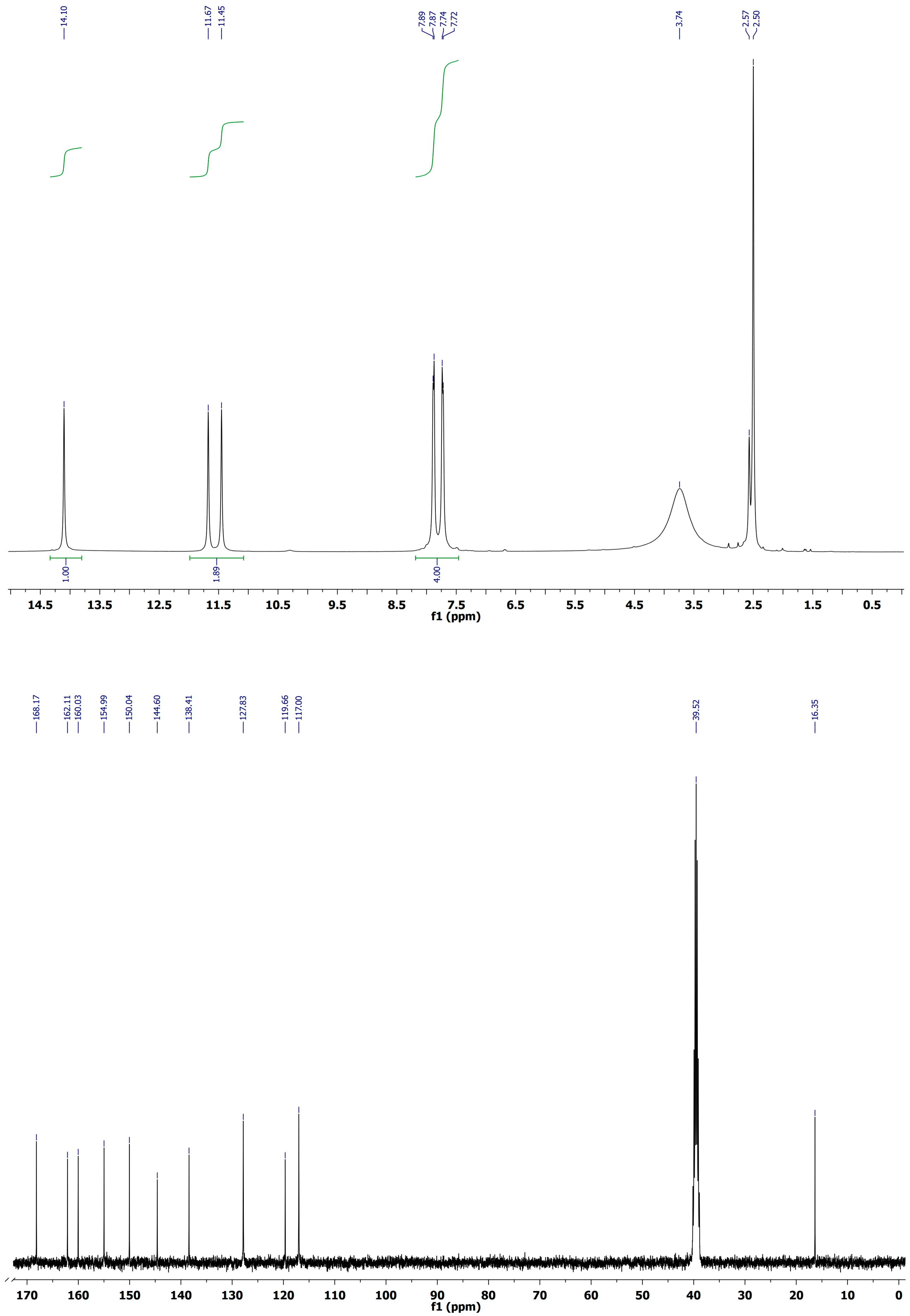

Figure S2. $^{1}H/^{13}C$ NMR spectra of **AL56.**

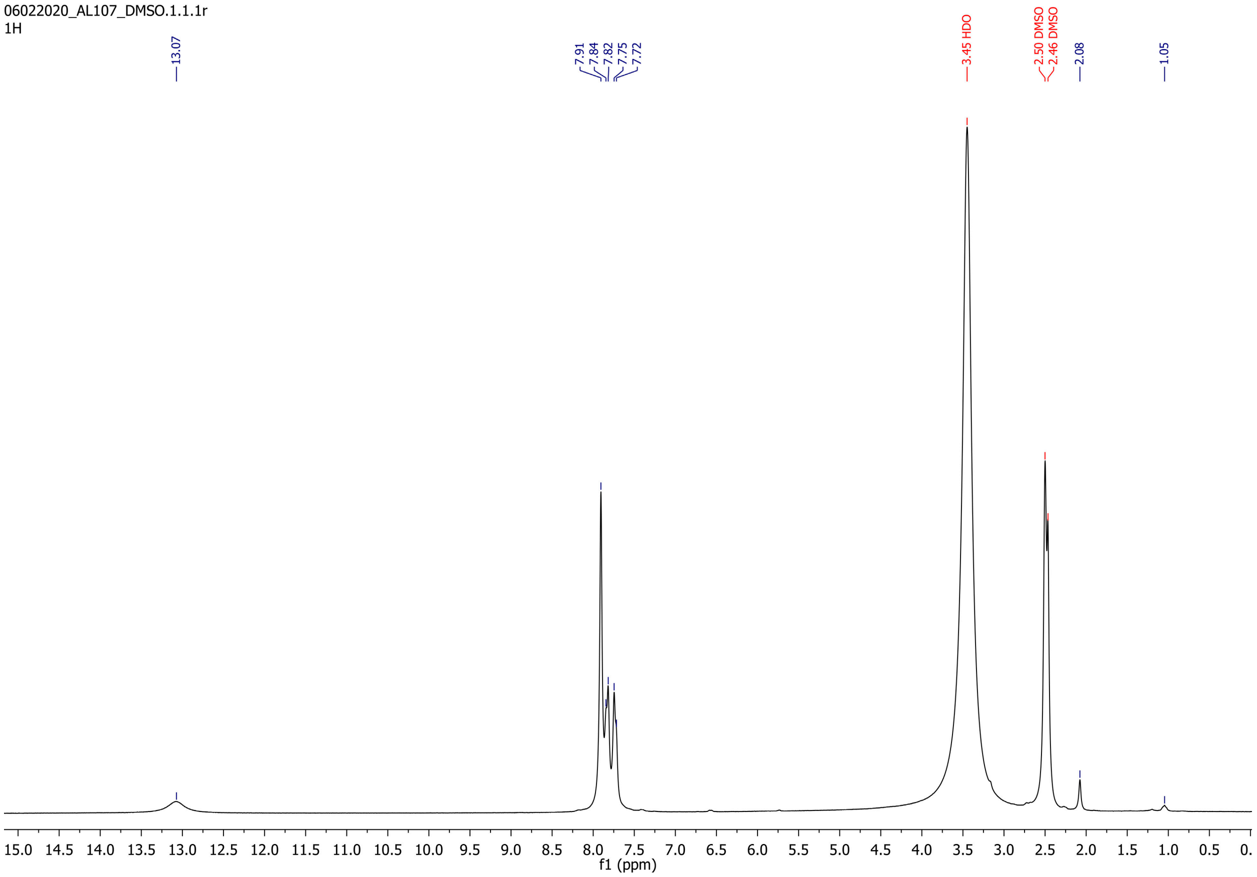

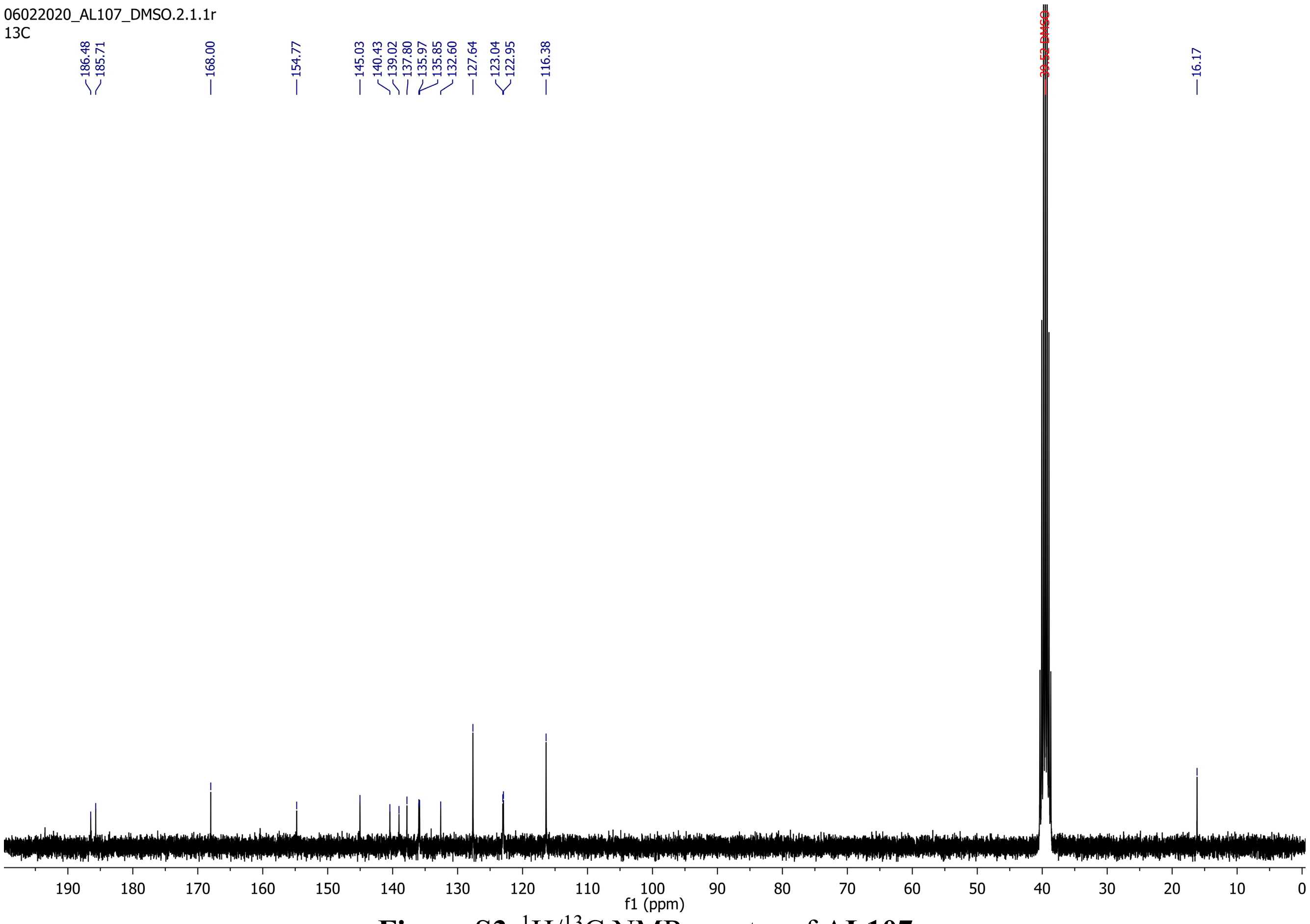

Figure S3. $^{1}H/^{13}C$ NMR spectra of **AL107.**

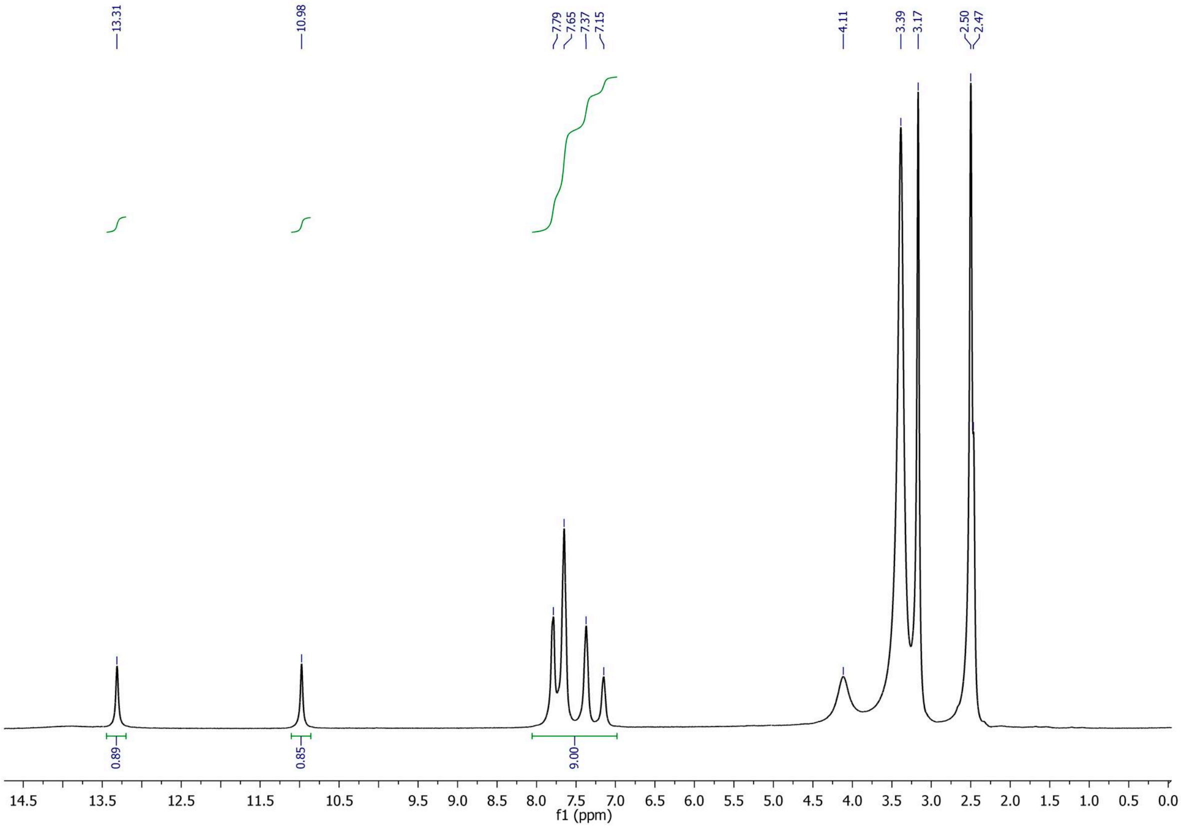

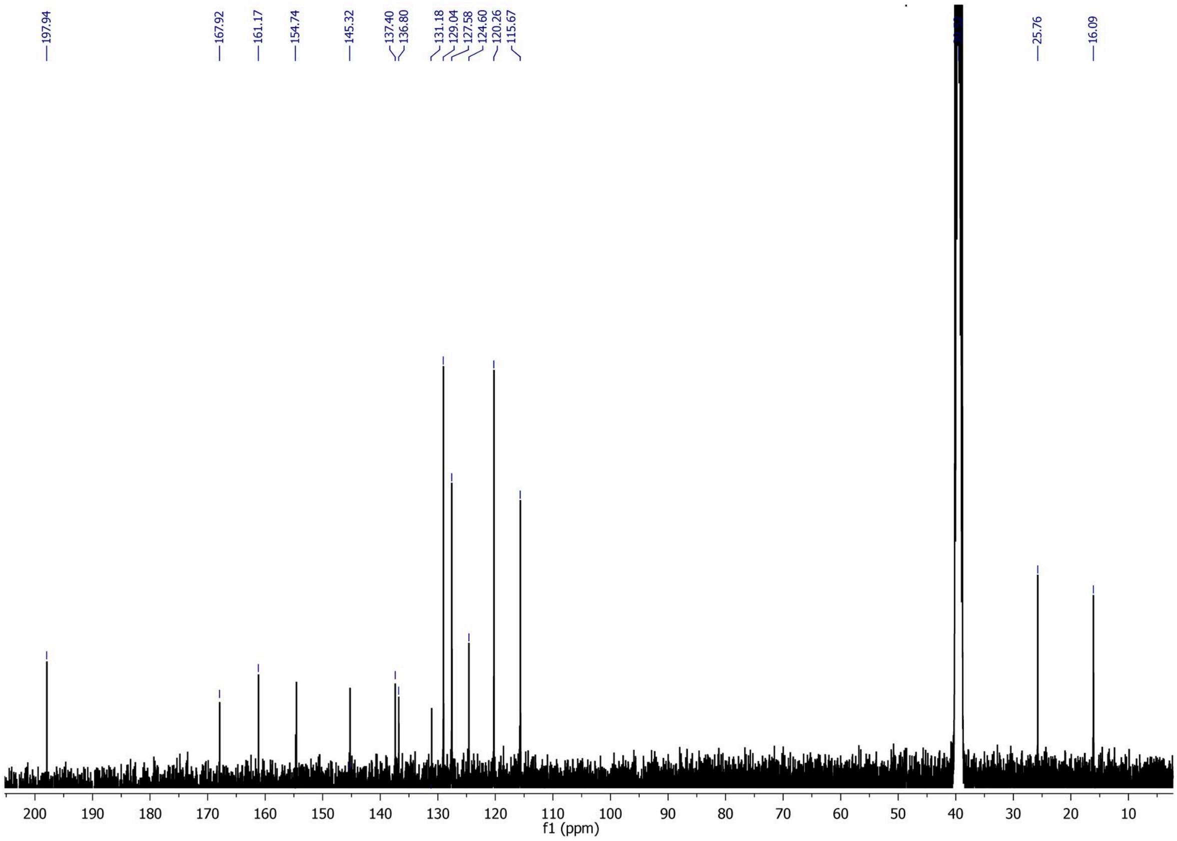

Figure S4. $^{1}H/^{13}C$ NMR spectra of **AL109.**

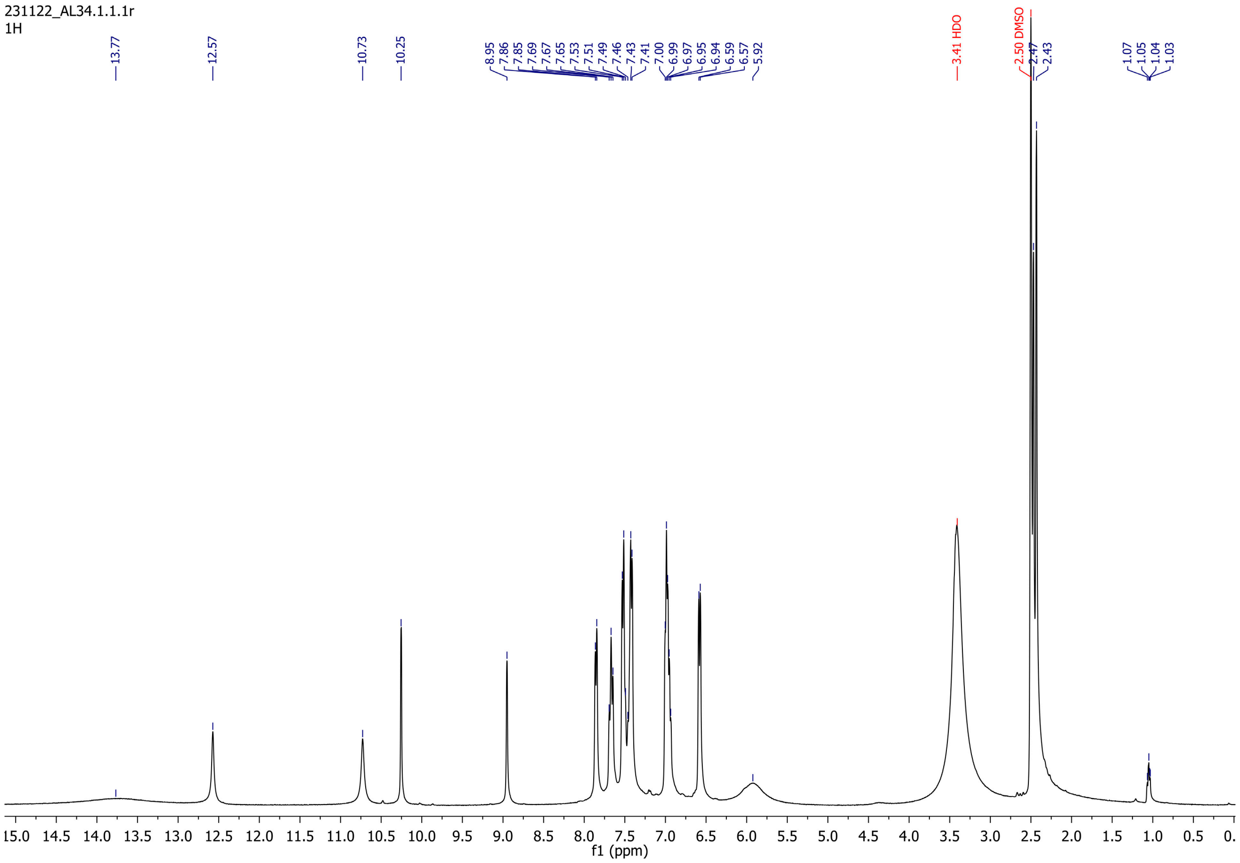

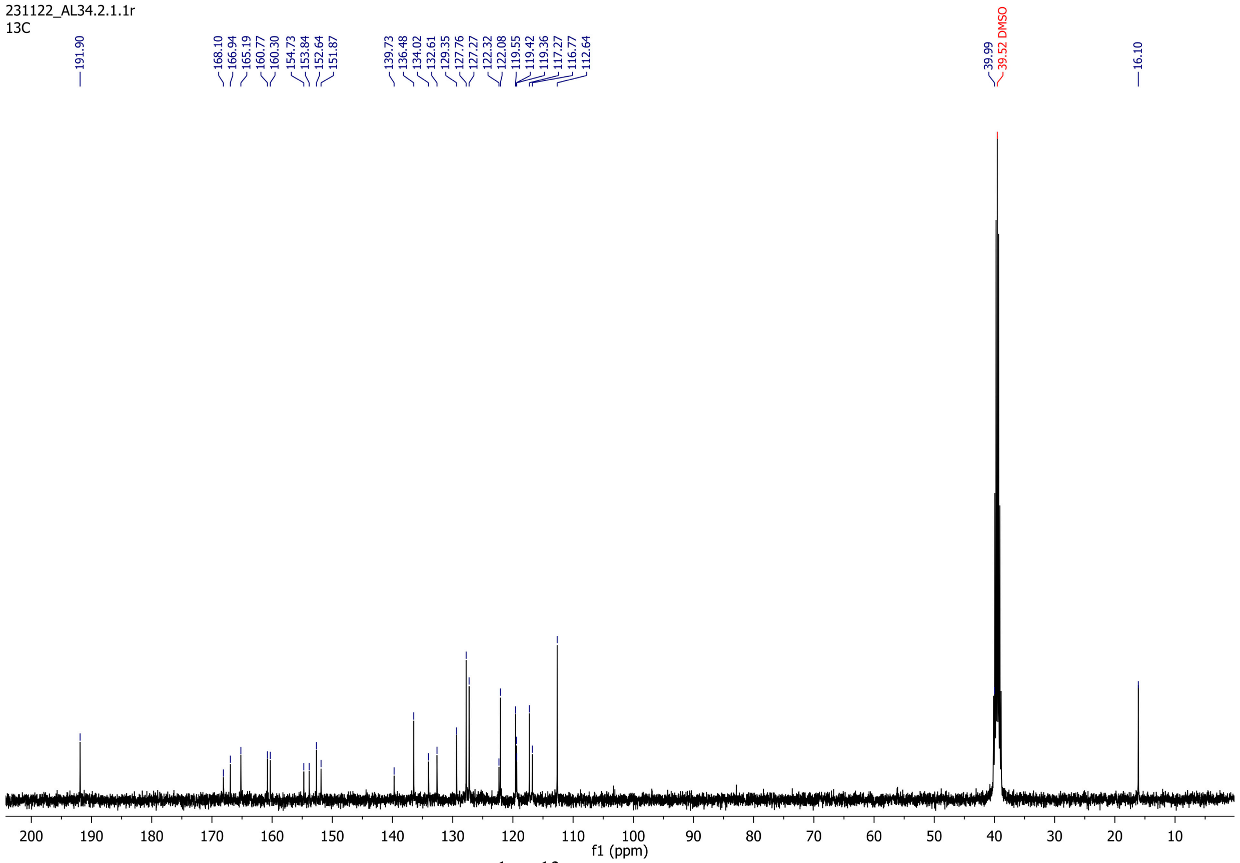

Figure S5. $^{1}H/^{13}C$ NMR spectra of **AL34.**

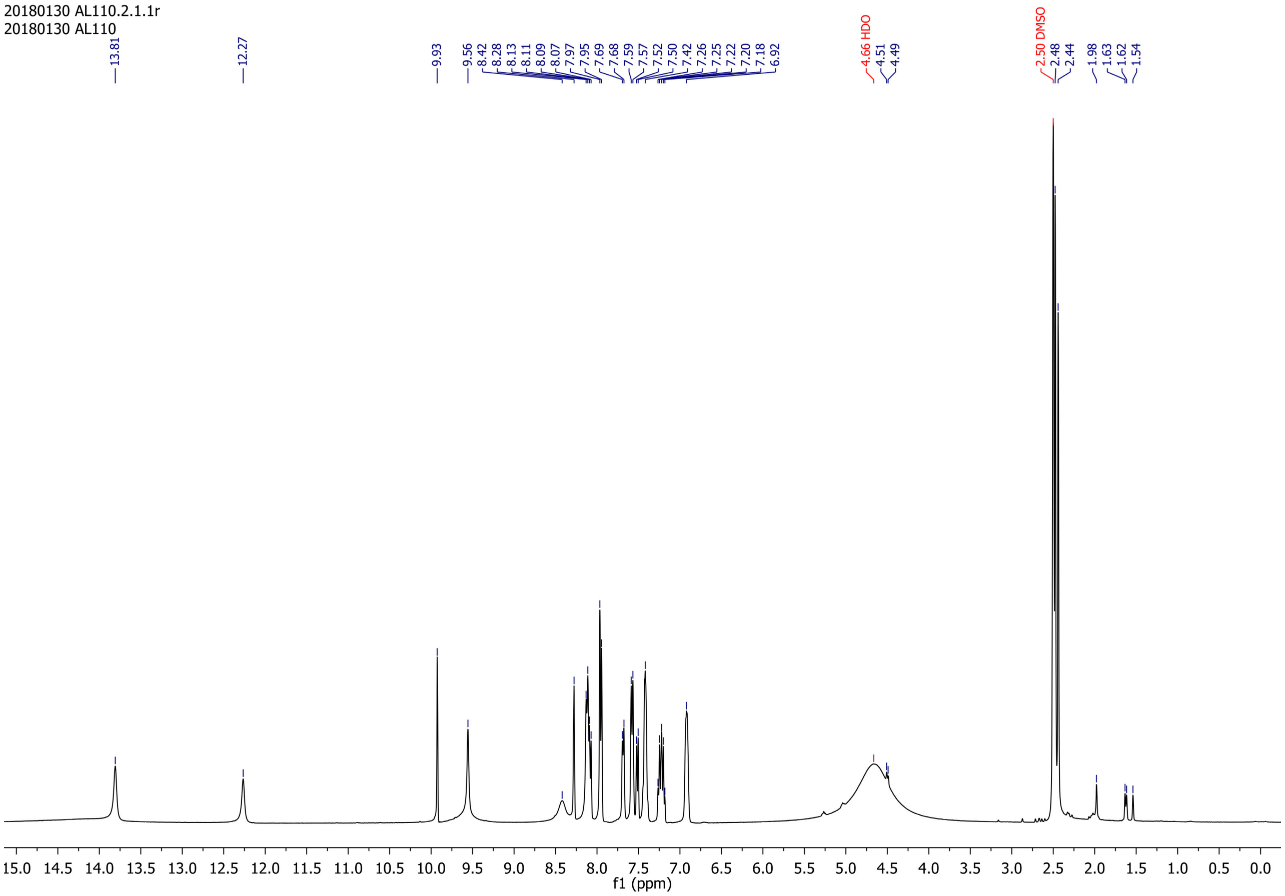

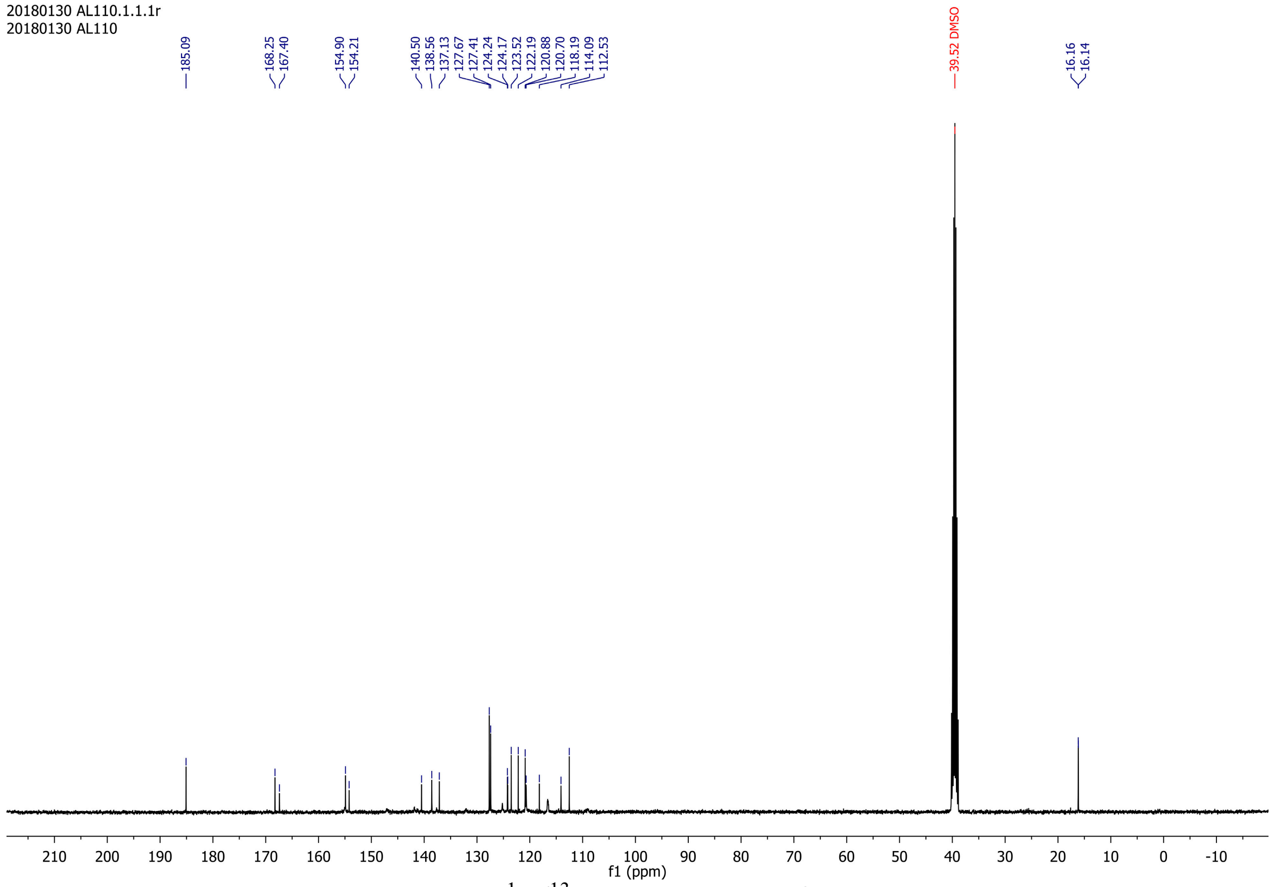

Figure S6. $^{1}H/^{13}C$ NMR spectra of **AL110.**
